# Online Adaptation of a c-VEP Brain-Computer Interface(BCI) Based on Error-Related Potentials and Unsupervised Learning

**DOI:** 10.1371/journal.pone.0051077

**Published:** 2012-12-07

**Authors:** Martin Spüler, Wolfgang Rosenstiel, Martin Bogdan

**Affiliations:** 1 Wilhelm-Schickard-Institute for Computer Science, University of Tübingen, Tübingen, Germany; 2 Computer Engineering, University of Leipzig, Leipzig, Germany; University of Adelaide, Australia

## Abstract

The goal of a Brain-Computer Interface (BCI) is to control a computer by pure brain activity. Recently, BCIs based on code-modulated visual evoked potentials (c-VEPs) have shown great potential to establish high-performance communication. In this paper we present a c-VEP BCI that uses online adaptation of the classifier to reduce calibration time and increase performance. We compare two different approaches for online adaptation of the system: an unsupervised method and a method that uses the detection of error-related potentials. Both approaches were tested in an online study, in which an average accuracy of 96% was achieved with adaptation based on error-related potentials. This accuracy corresponds to an average information transfer rate of 144 bit/min, which is the highest bitrate reported so far for a non-invasive BCI. In a free-spelling mode, the subjects were able to write with an average of 21.3 error-free letters per minute, which shows the feasibility of the BCI system in a normal-use scenario. In addition we show that a calibration of the BCI system solely based on the detection of error-related potentials is possible, without knowing the true class labels.

## Introduction

A Brain-Computer Interface (BCI) enables a user to control a computer by pure brain activity without the need for muscle control. Its main purpose is to restore communication in severely disabled people, who are not able to communicate by muscle activity due to neurodegenerative diseases or traumatic brain injuries. There are different kinds of BCIs, that are based on modulation of the sensorimotor rhythm (SMR), detection of a P300 or steady state visual evoked potentials (SSVEPs). In this paper we present a BCI that uses code-modulated visual evoked potentials (c-VEPs) to detect the user’s intention.

In a c-VEP BCI, a pseudorandom code is used to modulate different visual stimuli. If a person attends one of those stimuli, a c-VEP is evoked and thus can be used for controlling the BCI. This idea has been proposed by Sutter in 1984 [1] and has been tested 8 years later, when an ALS patient was reported to write 10 to 12 words/minute with a c-VEP BCI system using intracranial electrodes [2]. Until recently, there has been no proper evaluation of a c-VEP BCI with electroencephalography (EEG), when it was shown by Bin et al. [3] that a BCI based on c-VEPs outperforms BCIs based on other kinds of visual stimuli. In [4] and [5] new methods for improving classification in a c-VEP BCI were presented and the possibility for establishing high-performance communication was demonstrated.

In this paper, we evaluate the use of online adaptation to further improve a c-VEP BCI system. In a traditional BCI, a fixed amount of training data is collected and used to train a classifier, that remains unchanged throughout a session. By online adaptation of the classifier, new data that becomes available during the usage of the BCI can be used for continous training of the classifier and therefore reduce the amount of training data needed, while also improving performance by making the classifier more robust to changing data. The problem with online adaptation is the absence of true class labels. So the classifier can either be adapted in a completely unsupervised fashion or additional information, like the presence of error-related potentials (ErrPs), can be incorporated to improve adaptation.

Error-related potentials are event related potentials that can be detected shortly after the user recognizes an error. It has been shown in previous works [6], [7] that ErrPs can be classified with sufficient accuracy. It also has been shown that they can be used in a BCI to correct spelling errors and thereby could improve performance of a P300 BCI in healthy and severely disabled subjects [8]. That ErrPs can also be utilized for adaptation of a classifier has been recently shown in an offline study [9].

In this paper we show in an online study that adaptation increases performance in a c-VEP BCI and that ErrPs can be used online to improve adaptive classification. We also demonstrate the possibility of c-VEP BCIs to establish high-performance communication. In addition we show that a calibration of the BCI-system solely based on the detection of ErrPs is possible.

## Methods

### Configuration of the c-VEP BCI System

The c-VEP BCI system is similar to the one described in [4], consisting of an EEG amplifier, a personal computer (PC) and a CRT Monitor. Stimulus presentation and online classification are operated from the PC. The presentation of the stimuli are synchronized with the EEG amplifier by using the parallel port. BCI2000 [10] is used as a general framework for recording the data. The visual stimuli are presented on a 17 inch CRT Monitor with a 60 Hz refresh rate and a resolution of 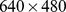 pixel. The subjects are seated approximately 80 cm in front of the monitor. To ensure synchronization of the presented stimuli with the refresh rate of the CRT monitor, DirectX (Microsoft Inc.) is used for programming the stimulation module.

A stimulus can either be black or white, which can be represented by 0 or 1 in a binary sequence. A 30 Hz flickering can therefore be represented by the following sequence : ‘01010101…’ when using a 60 Hz refresh rate.

**Figure 1:**
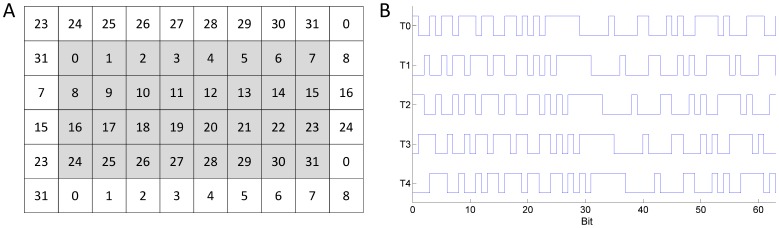
Arrangement and modulation of the stimuli for the c-VEP BCI. **A:** The gray area shows the 32 target stimuli with the number referring to the number of the target. The stimuli in the white area are the complementary flickers, which are synchronized to the target with the same number. **B:** Modulation sequence for the first 5 targets. The sequence of a target is shifted by 2 bit in respect to its preceding target.

The c-VEP BCI consists of 32 targets with the arrangement of the targets shown in figure 1. The 32 targets are arranged as a 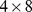 matrix and 28 complementary non-target stimuli are surrounding the targets. For modulation of the targets a 63-bit binary m-sequence is used, because of the low auto-correlation property of m-sequences [11]. For each target the same sequence is used for modulation, but the sequence is circular-shifted for each target by a different number of bits. An example for the circular shift of the modulation sequence can be seen in figure 1, with target 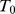 having no shift, 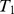 being shifted by 2 bit, 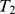 being shifted by 4 bit and so on, resulting in a time lag 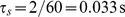 between two consecutive targets. In total the length of one stimulation sequence is 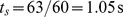. Between two stimulation sequences there is a break of about 0.85 s which is sufficient enough for the user to switch his attention to a different target (i.e., look at a different target).

**Figure 2:**
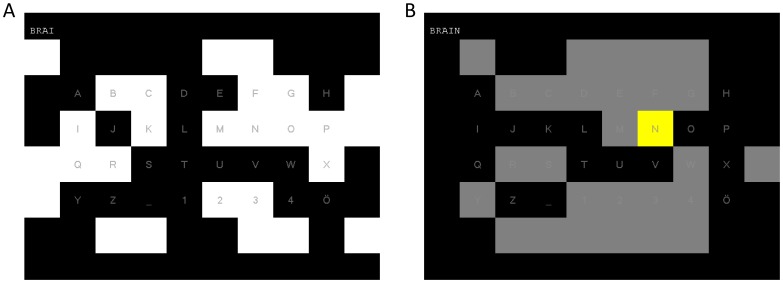
Screenshots of the c-VEP BCI in free-spelling mode. **A:** screenshot during a trial. The letter Ö (lower right corner) serves as a backspace symbol during the free-spelling condition and allows the user to correct mistakes. **B:** screenshot of the letter N being selected and the other characters being grayed out to indicate a selection.

In our system the 32 targets were used to select one of the 26 letters A to Z from the alphabet as well as underscore and the numbers 1 to 5. In the free-spelling condition the number 5 was replaced with the character Ö, which was used as a backspace. A screenshot of the matrix that was displayed to the subjects can be seen in figure 2. If a target was selected, the corresponding character was written and it was indicated to the user by highlighting the selected target in yellow for 150 ms and darkening the rest of the matrix for the same time, so that the user is also aware that a selection has happened if he looks on a different part of the screen. The text that has been written by the user is displayed on the top of the screen.

### Calibration and Classification

The calibration of the c-VEP BCI is done in 3 steps. First, training data needs to be collected. Second, spatial filter are generated by CCA based on the training data. In a third step, the classifier is trained by generating templates.

As already mentioned, before calibrating the c-VEP BCI system, training data needs to be collected. Therefore the user has to attend a given target 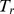 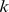 times, i.e., the user has to look at a specified letter on the matrix (associated with 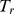) for 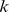 trials. 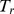 can be chosen arbitrarily with 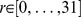. The result are 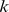 trials with each trial consisting of EEG-data with dimensions 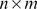, where 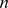 is the number of channels and 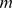 is the number of samples during a trial.

For the generation of spatial filters, first ones needs to find the channel 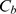, for which the c-VEP is most prominent. Therefore, a leave-one-out cross-validation is performed: For each trial, templates are generated by averaging over the remaining trials (and shifting, as will be explained later) and the template with the highest correlation with the tested trial is selected. To estimate the accuracy for one channel, the percentage of correctly selected templates is calculated. This is done for all channels and the channel with the highest estimated accuracy is selected as 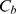.

Canonical Correlation Analysis (CCA) [12] is then used for generation of a spatial filter. The goal of CCA is to find linear transformations 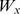 and 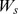, which maximize the correlation between 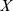 and 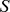:
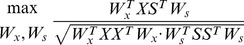
(1)


To obtain an optimal spatial filter 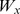, 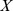 is the raw EEG-data and 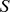 is the desired waveform of the average c-VEP. To generate 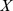, all 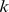 trials are concatenated to a new matrix 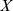 with the dimensions 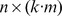.

To generate 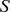, the EEG data from channel 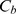 is used and by computing the average of all 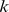 trials the average c-VEP waveform 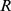 for channel 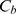 is obtained, with 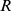 having the dimensions 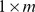. As a next step, 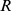 is replicated 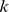 times, leading to 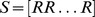 with dimensions 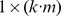. Having 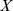 and 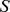, CCA can be applied and the resulting 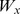 can then be used as a spatial filter and multiplied with the raw EEG-data, to obtain spatially filtered EEG-data.

To train a classifier, we use a one class support vector machine (OCSVM) [13], which we have shown to be superior to the classical correlation approach [5]. The OCSVM is trained with the spatially filtered training data. The result of the OCSVM is a hyper-sphere with minimal radius, that encloses a given percentage of the data. The center of the hyper-sphere can be used as a template 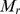, that represents the evoked response for attending target 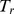. From another point of view, the use of a OCSVM can be seen as a more robust method for averaging that rejects outliers. Since all targets are modulated with the same code, but different shifts, templates for all other targets can be generated by shifting the template 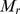:
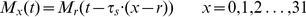
(2)


For classification of a new trial with unknown label, the euclidean distance between the spatially filtered EEG data and all templates is calculated, the template with the smallest distance to the EEG data is found and the corresponding target is selected. For implementation of the OCSVM we used LibSVM [14] with a linear kernel and 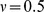.

#### Classifier calibration through supervised adaptation

The classical approach to train a BCI system is to collected training data without giving the user feedback and train the classifier after all training data is collected. We employed a co-adaptive calibration approach similar to [15], in which feedback is given during the calibration right from the beginning. The system starts with randomly generated templates as classifier and the classifier is adapted in a supervised manner to calibrate the BCI system. Since the correct target class is known for each trial during calibration, it is not necessary to just use target 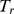 during calibration, but different targets can be used. Data obtained when attending 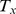 can be circular-shifted to fit the shift of 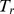, added to the training data and thus be used to calculate spatial filters by CCA and train the OCSVM.

#### Unsupervised classifier adaptation

**Figure 3:**
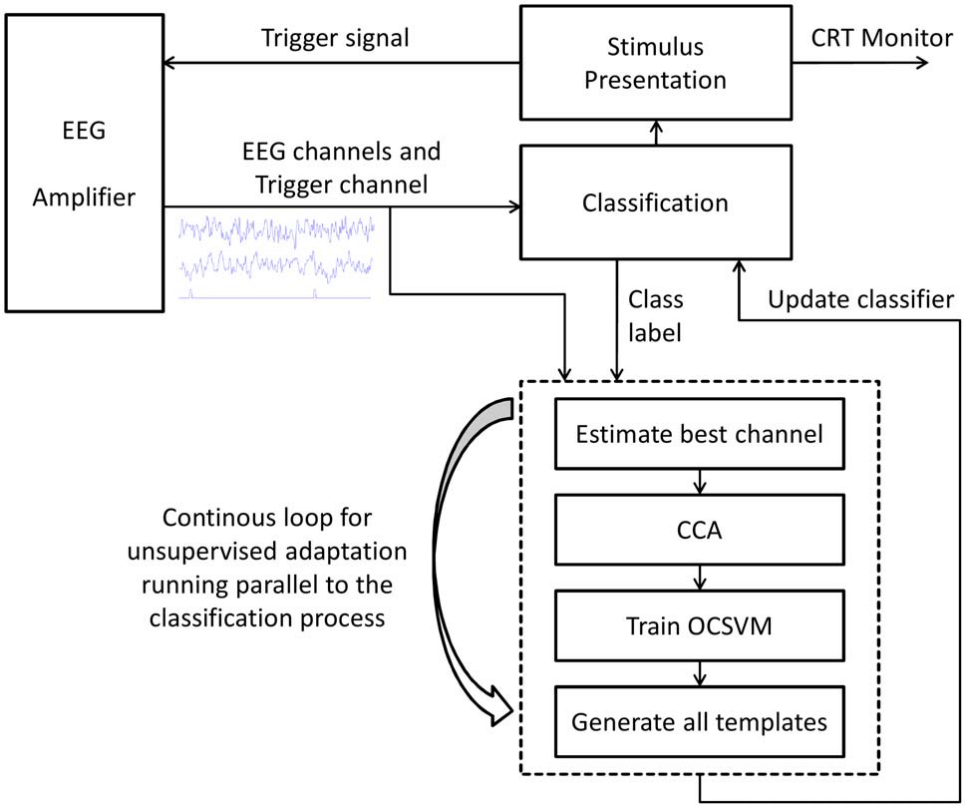
Diagram of the c-VEP BCI system with unsupervised adaptation. The process for adaptation of the classifier is running in a loop parallel to the classification process. Both processes are communicating via shared memory.

While the true target is known for the supervised adaptation during the calibration of the c-VEP BCI system (target is given and known to the user), the true target is unknown when using the system after it has been calibrated (when the user can freely decide what to write). To further improve classification after calibration is finished, the BCI is adapted in an unsupervised manner. For a new Trial 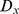, the trial is classified resulting in a estimated label 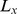. 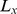 is assumed to be the correct class label and the classifier is adapted by adding 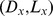 to the training data and retraining the classifier. Retraining the classifier involves estimation of the best channel, generating spatial filter by CCA, training a OCSVM and generating templates for all targets. A diagram that visualizes the c-VEP BCI system with unsupervised adaptation is shown in figure 3.

The adaptation of the classifier is done in a loop parallel to the signal processing and classification module of BCI2000. Communication between both modules is done via shared memory. If new EEG data arrives during the adaptation process, it is stored in a buffer and used for adaptation in the next iteration of the adaptation loop.

#### ErrP-based classifier adaptation

In addition to the unsupervised adaptation, ErrPs can be utilized to detect misclassifications. If no ErrP is detected, the data is used for unsupervised adaptation as explained before. If an ErrP is detected, the data is not used for adaptation of the classifier since the true class label is unknown and the estimated class label is suspected to be wrong.

#### ErrP-based calibration

If only 2 targets are available (e.g., targets J and W), ErrPs can also be used for calibration and thereby make it possible to omit a supervised calibration. At the beginning of the ErrP-based calibration the classifier starts with randomly generated templates. Each new trial 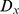 is classified resulting in a corresponding label 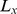. The result is displayed to the user and the time period after displaying the result is used for detection of an ErrP. If no ErrP is detected, 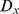 is used for adaptation of the classifier with the corresponding label 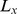. If an ErrP is detected, 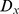 is used for adaptation with the opposite label of 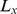 (W if 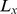 is J and vice versa). To compare the accuracy with the supervised adaptation, a supervised calibration was simulated offline.

Due to the design of the c-VEP system based on the circular shift of the modulating code, a calibration with 2 targets is sufficient and the data can be used for generating templates for all 32 targets.

#### Detection of error-related potentials

For classification of the ErrPs we basically used the same procedure as we already described in [8] where ErrPs in a P300 BCI were detected: The signal was re-referenced to the common average, linear trends were removed, it was bandpass-filtered between 1 Hz and 16 Hz and subsequently resampled to 32 Hz. For the classification of the ErrP in this study we used the time interval between 300 ms and 990 ms after the selection of a target. This time interval seemed to be best in a not representative one-subject experiment that was performed with the c-VEP BCI prior to the study presented in this paper. The time domain samples of channels Fz,Cz,Cpz,Pz and POz were used for classification. Classification was done with LibSVM [14] using a RBF-Kernel with default parameters (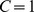, 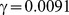).

### Design of the Online Experiment

To test the system with unsupervised and ErrP-based adaptation, 10 healthy subjects were recruited. All subjects had normal or corrected-to-normal vision. A summary over age, sex and previous BCI experience of the subjects can be found in table 1. The study was approved by the local ethics committee of the Medical Faculty at the University of Tübingen. Written consent was obtained from all subjects. Each subject participated in two sessions. For 4 subjects session 1 and session 2 were performed on different days, since they also participated in another EEG study that was done on two different days. For the other subjects both sessions were performed on the same day with a break of about 10 minutes.

During the preparation of the EEG setup subject AJ reported having problems with her contact lenses the previous days. After several unsuccessful tries to perform a proper calibration session, subject AJ was excluded from the study. Due to excessive blinking it was not possible for her to follow all cues during the calibration session, which resulted in attending the wrong targets.

**Table 1:** Subject overview.

				previous BCI experience			
Subject	Age	Sex	Days between	c-VEP	SSVEP	SMR	P300
AA	26	f	1	–	–	x	x
AB	29	f	3	–	–	x	–
AC	28	m	1	–	–	x	x
AD	26	f	0	–	–	x	x
AE	29	m	0	–	–	x	–
AF	28	m	1	–	–	x	–
AG	28	m	0	–	–	o	–
AH	28	m	0	x	–	x	–
AI	28	m	0	–	–	–	–
AJ	28	f	–	–	–	–	–

Age and sex of the subjects as well as the number of days between session 1 and session 2 and the previous BCI experience of the subject: **-** subject has no experience with that kind of BCI, **x** subject has previously used that kind of BCI and was able to control it, **o** subject has previously used that kind of BCI but was not able to control it. Subject AJ was excluded from the study due to excessive blinking.

**Figure 4:**
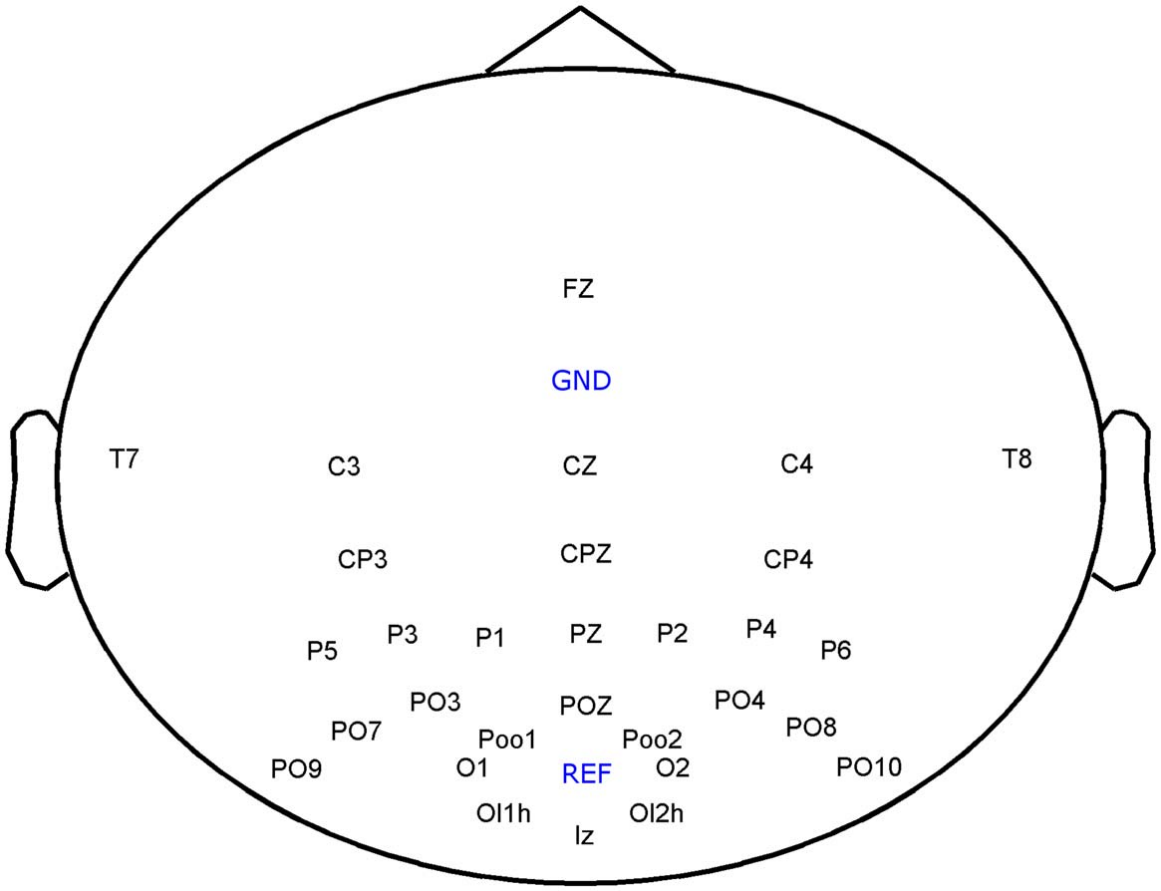
Location of the 30 EEG electrodes. Ground electrode (GND) was positioned at FCz and reference electrode (REF) at Oz.

EEG data was recorded with a g.tec g.USBamp at a samplingrate of 600 Hz and a Brainproducts Acticap system with 32 channels. Two electrooculogram (EOG) electrodes were placed beside the left eye and at the center above the eyes. The location of the 30 EEG electrodes is depicted in figure 4. The ground electrode was positioned at FCz and the reference electrode at Oz. The data was bandpass-filtered by the amplifier between 0.5 Hz and 60 Hz using a Chebyshev filter of order 8 and an additional 50 Hz notch filter was applied.

At the the beginning of the first session, a supervised calibration was performed. As previously mentioned, the BCI was calibrated in a co-adaptive manner by supervised adaptation and giving feedback during the calibration. The calibration consisted of 64 trials with each of the 32 letters being spelled twice. After calibration, the unsupervised adaptation was tested in 9 runs with 64 trials each (total of 576 trials). The unsupervised adaptation was tested in a copy-spelling mode, in which it was given to the user which letters he had to write.

At the beginning of the second session, a supervised calibration of the BCI was performed similar to session 1. After calibration, 9 runs in a copy-spelling mode with 64 trials each (total of 576 trials) were performed to test ErrP-based adaptation.

Independent of this, at the end of the second session, some subjects participated in additional experiments, in which either an ErrP-based calibration was tested or the subjects used the c-VEP BCI in a free-spelling mode (details on this will be described later).

### Performance Evaluation

To compare the results from different sessions and for the different adaptation methods, the accuracy of the classifier, as well as the corresponding information transfer rate (ITR) [16] were used. With 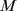 being the number of classes and 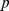 being the accuracy, the ITR can be computed with the following equation:
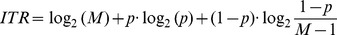
(3)


Although the ITR is a commonly used measure for BCI performance, that allows for a good comparison of different BCI systems, it is a rather theoretic approach for assessing the BCI performance that does not take into account the actual design of the BCI application and therefore tends to misestimate the real BCI performance [8], [17]. To assess the real performance of the BCI as a spelling application we used the average number of correct letters per minute in the free-spelling condition, taking into account that all errors are corrected by the user.

### Offline Data Analysis

To compare the results for unsupervised and ErrP-based adaptation, a comparison of the results from session 1 and session 2 would be deceiving, because of different additional factors influencing the data and thus the BCI performance. Instead we used the data from session 2 to simulate online experiments with different kinds of adaptation. Exactly the same data was used for calibration and testing, but different adaptation methods were employed during the test runs. We tested without adaptation, with unsupervised adaptation and supervised adaptation. For the supervised adaptation we used the real label of the target, which would not be available when using the BCI as intended in a free-spelling mode.

### Online ErrP-based Calibration

To test if a calibration without known class labels is possible, the detection of ErrPs should be used for calibration. The chronologically last 4 subjects of the study(AA,AD,AG,AI) also participated in an additional online experiment to test ErrP-based calibration. Although the stimulus presentation was the same as the one described before with 32 targets, only 2 targets (letter J and letter W) should be used during the calibration. In contrast to the calibration described before, the subject could freely choose between fixating the letter J or the letter W this time. They were only instructed not to switch the target every trial and not to stay at the same target for longer than 5 trials. Since an afterwards evaluation of the c-VEP classification accuracy during the calibration is difficult with these instructions, ErrP-based calibration was also performed with the instruction to start at letter J and switch the target every trial. Only results from the data recorded with the latter instruction are shown in this paper. The classification during the calibration period could only result in the labels of the two targets corresponding to letter J and W.

### Online Free-spelling

To assess the performance of the c-VEP BCI under normal-use conditions, some of the subjects engaged in free-spelling at the end of session 2. At this point each of the participating subjects had about 1 hour of total experience with the c-VEP BCI system. Target 32 was replaced with the letter Ö, which served as a backspace option and allowed the user to delete the previous letter. The subjects could write whatever they felt like and they were only instructed to correct each mistake by choosing the backspace symbol.

## Results

### Online Experiment using Unsupervised and ErrP-based Adaptation

The results from the online experiment can be seen in table 2. During session 1 with unsupervised adaptation the subjects achieved an average accuracy of 92.53%, which corresponds to an average ITR of 135.62 bit/min. During session 2 with ErrP-based adaptation an average accuracy of 96.18% was achieved, which corresponds to an average ITR of 143.95 bit/min. It should be noted that subject AG and subject AD achieved 100% accuracy in one of the sessions (576 trials).

**Table 2:** Results from the online experiment.

	Session 1 (unsupervised)		Session 2 (ErrP-based)	
Subject	Accuracy	ITR [bit/min]	Accuracy	ITR [bit/min]
AA	98.78%	151.53	97.40%	147.16
AB	86.28%	119.05	91.49%	130.78
AC	98.44%	150.61	97.05%	145.55
AD	99.13%	152.52	100.00%	156.28
AE	77.26%	96.94	99.83%	155.06
AF	96.70%	144.79	94.27%	137.76
AG	100.00%	156.23	99.48%	153.80
AH	78.99%	102.50	89.93%	126.09
AI	97.22%	146.44	96.18%	143.03
**average**	**92.53%**	**135.62**	**96.18%**	**143.95**

Accuracy and corresponding information transfer rate for the 9 subjects during the online experiment with unsupervised adaptation (session 1) and with adaptation based on Error-related potentials (session 2).

### Offline Data Analysis

To compare the effect of unsupervised and ErrP-based adaptation we performed an offline analysis, in which the online experiment was simulated with the same data, but different methods for adaptation. The results are shown in table 3. Without adaptation, using only the first calibration run for training the classifier, an average accuracy of 95% was achieved, which corresponds to an average bitrate of 140.46 bit/min. With unsupervised adaptation an average accuracy of 96.05% was achieved, which corresponds to an average bitrate of 143.56 bit/min, while the online results with adaptation based on ErrPs yielded an average accuracy of 96.18% or 143.95 bit/min. The simulation with supervised adaptation resulted in an average accuracy of 97.00%, which corresponds to a bitrate of 146.47 bit/min.

**Table 3:** Offline results from session 2 with different adaptation methods.

Subject	No adaptation	Unsupervised	ErrP-based	Supervised
AA	94.44%	98.44%	97.40%	98.44%
AB	87.48%	88.60%	91.49%	92.78%
AC	98.09%	98.09%	97.05%	98.09%
AD	100.00%	100.00%	100.00%	100.00%
AE	99.31%	99.83%	99.83%	99.83%
AF	94.97%	95.14%	94.27%	95.83%
AG	98.96%	99.48%	99.48%	99.48%
AH	86.98%	88.54%	89.93%	91.15%
AI	94.79%	96.35%	96.18%	97.40%
**average**	**95.00%**	**96.05%**	**96.18%**	**97.00%**

Accuracies with different adaptation methods on the data from session 2. The condition without adaptation, with unsupervised adaptation and with supervised adaptation were simulated offline with the data from session 2. The results for the ErrP-based adaptation are the online results from session 2.

While the results with unsupervised and ErrP-based adaptation are significantly better than the results with no adaptation (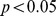, paired t-test), there is no significant difference between unsupervised and ErrP-based adaptation (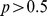, paired t-test).

To evaluate the benefit of the different preprocessing and classification methods without adaptation, we also performed an additional offline analysis of the data from session 2. When using the method by Bin et al. [4] an average accuracy of 88.48% was achieved. Using a OCSVM [5] instead of the correlation approach yielded an average accuracy of 91.99%. When combining OCSVM with a different method of applying CCA by selecting the best individual channel [5] an accuracy of 95.00% was reached, which is the method we used online and also described in the methods section.

### Details on Code-modulated VEPs

**Figure 5:**
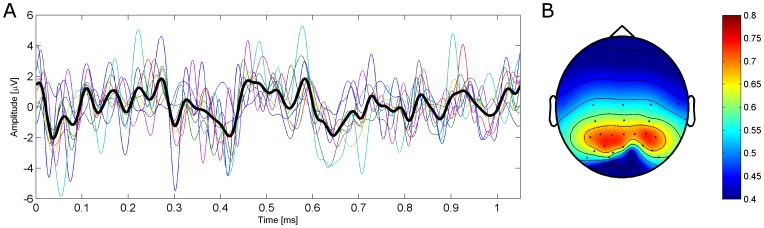
Average c-VEP waveform for target 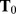 A: Average waveform of the elicited c-VEP at electrode P4. The subjects’ average c-VEP are depicted by the colored lines. The average c-VEP over all subjects is shown by the black bold line. **B:** Estimated accuracy for one channel averaged over all subjects. Accuracy was estimated with the same method that was previously used for finding the best channel 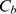.

To see, on which channel the c-VEP is strongest and can be classified best, classification accuracies were estimated by using only one channel. The accuracies have been estimated for each subject separately with a leave-one-out cross-validation without the use of CCA by just using the classical correlation approach [4] for classification. It shows that the accuracy is highest at electrodes P4 and PO3 with an average accuracy of 73.2% and 72.6%, respectively. The average accuracy for each channel is shown in figure 5. The average c-VEP waveform on the subject’s individual best channel is also shown in figure 5.

To estimate the delay of the c-VEP, the cross-correlation of the average c-VEP with the modulation sequence was computed. It was highest for a 36 ms delay of the c-VEP with 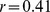.

### ErrP-based Calibration

Normally, calibration is done supervised and therefore no accuracies and bitrates are presented. But in contrast to a supervised calibration, the user can transfer information during a ErrP-based calibration and therefore classification accuracies and corresponding information transfer rates are of interest. During the ErrP-based calibration, on average 85.94% of the targets were classified correctly, which corresponds to an average bitrate of 18.28 bit/min (taking into account that only 2 targets can be chosen). For subject AD no ErrPs were detected and therefore she achieved an average accuracy of 43.75%, which is below chance level (50%). An overview of the results for the c-VEP classification during the ErrP-based calibration is shown in table 4.

**Table 4:** Results for the c-VEP classification during the ErrP-based calibration.

Subject	Errors	Correct	Trials	Accuracy	ITR [bit/min]
AA001	12	116	128	90.63%	17.24
AA002	2	126	128	98.44%	27.42
AD001	72	56	128	43.75%	0
AG001	2	62	64	96.88%	25.21
AG002	3	61	64	95.31%	22.56
AI001	12	116	128	90.63%	17.25
**average**				**85.94%**	**18.28**

Number of erroneus, correct and total trials, accuracy and corresponding bitrate for the c-VEP classification during the 6 ErrP-based calibrations. For 2 subjects (AA, AG) 2 ErrP-based calibrations were performed.

**Figure 6:**
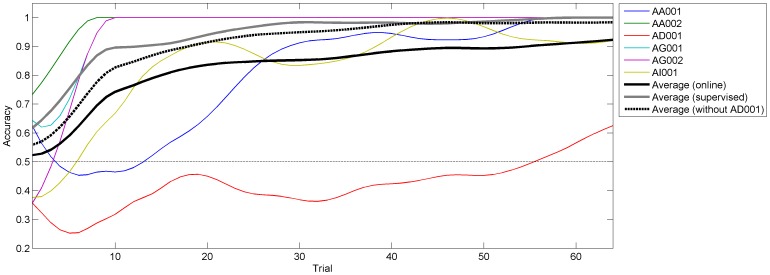
Accuracy for the c-VEP classification during the ErrP-based calibration. The plot shows the accuracy over the first 64 trials. For better presentation, the data is smoothed. The colored lines depict the smoothed accuracy for each of the 6 ErrP-based calibrations. The black solid line is the average over all calibrations. The black dashed line is the average over all calibrations excluding AD001. The gray line is the average over all calibrations simulated with supervised adaptation.

In figure 6 the c-VEP classification accuracy over the course of the 64 trials of the calibration is shown. For better viewing the data was smoothed. It can be seen, that during 3 calibrations (AA02, AG001, AG002) a near perfect accuracy was reached with no errors being made after the 10th trial. With exception of subject AD, sufficient accuracy was reached after 25 trials, which takes less than 1 minute. The dashed bold black line in figure 6 depicts the average accuracy excluding subject AD. It can be seen that this average accuracy is close to the average accuracy obtained by the simulated calibrations with supervised adaptation (depicted by the gray line in figure 6), showing that an ErrP-based calibration can reach similar accuracies as a supervised calibration.

The ErrP-based calibration was also tested with the subject’s being instructed to freely choose the letters, but due to the nature of this instruction, we can not show accuracies for calibrations with this instruction. It still should be mentioned that the subjects perceived no difference in the accuracy of both methods.

The classifier obtained during the ErrP-based calibration was not tested with 32 targets, but due to the design of the c-VEP BCI with its circular-shifted code, the calibration on two targets is enough to use the c-VEP BCI system with 32 targets. Prior to this study, we tested the a classifier based on a supervised calibration with 2 targets on a system with 32 targets. One subject participated in this non-representative test, and achieved an accuracy of 100% over 64 trials, which shows that calibration on 2 targets is sufficient to use the system with 32 targets.

### Details on Error-related Potentials

The accuracies for detecting the ErrPs during the ErrP-based adaptation can be seen in table 5, with an average accuracy of 96.67% and an average sensitivity of 69.31%. The sensitivity refers to the percentage of ErrPs correctly identified, while specificity refers to the percentage of trials without ErrP that are classified correctly. The accuracies for detecting ErrPs during the ErrP-based calibration can be seen in table 6, where an average accuracy of 86.2% was achieved with a sensitivity of 45.83%. It should be noted that for subject AD no ErrP was classified although 43.75% of the trials were erroneus. There is a negative correlation between the number of ErrP trials in the data used for training the classifier and the sensitivity of the ErrP-detection (spearman 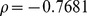, 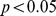).

**Table 5:** Results for the ErrP detection during ErrP-based adaptation.

Subject	Sensitivity	Specificity	Accuracy
AA	53.33%	99.11%	99.12%
AB	90.57%	93.33%	93.85%
AC	64.71%	97.05%	98.96%
AD	–	100.00%	100.00%
AE	100.00%	95.65%	95.66%
AF	36.36%	99.26%	95.66%
AG	0.00%	98.25%	97.74%
AH	74.14%	94.98%	92.88%
AI	77.27%	99.28%	98.44%
**average**	**69.31%**	**97.77%**	**96.67%**

Sensitivity, specificity and accuracy of the ErrP detection during the ErrP-based adaptation. Since the BCI worked with 100% accuracy for subject AD there is no sensitivity to report.

**Table 6:** Results for the ErrP detection during ErrP-based calibration.

Subject	Sensitivity	Specificity	Accuracy
AA001	25.00%	96.55%	89.84%
AA002	100.00%	97.62%	97.66%
AD001	0.00%	100.00%	43.75%
AG001	50.00%	100.00%	98.44%
AG002	66.67%	98.36%	96.88%
AI001	33.33%	96.55%	90.63%
**average**	**45.83%**	**98.18%**	**86.20%**

Sensitivity, specificity and accuracy of the ErrP detection during the ErrP-based calibration.

It should be mentioned, that with a subject-wise cross-validation, where the ErrP for one subject is classified based on the data of the remaining subjects, an average accuracy of 93.67% with a sensitivity of 57.57% and a specificity of 95.11% was achieved. For none of the subjects the performance was increased by the subject-wise cross-validation.

**Figure 7:**
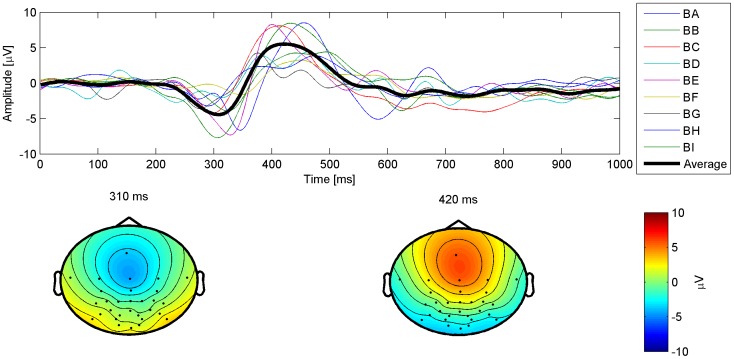
Average ErrP waveform. Error-minus-correct time series at electrode Cz for all subjects and the average as well as the topographical distribution at the time of the 2 peaks averaged over all subjects. The data was corrected for EOG [18] and bandpass-filtered between 1 Hz and 16 Hz.

Figure 7 shows the average error-minus-correct plot for the electrodes Fz, Cz, Cpz, Pz as well as the topographic distribution of the ErrP at two time points. It can be seen that the ErrP has two main components: a small negative peak at around 310 ms and a positive peak at 420 ms. Both peaks are most prominent between electrodes Fz and Cz.

### Free-spelling Results

The results from the 6 subjects who participated in the free-spelling can be seen in table 7. In total 603 trials were used to spell 427 error-free letters. 88 characters were wrong and the delete-character therefore has been chosen 88 times. The delete-character has never been selected erroneously and was selected with an accuracy of 100%. Considering all errors were deleted and considering the time needed to write a letter an average of 21.35 correct letters could be written per minute. Due to the fact that these results were obtained in a free-spelling condition, in which each error was corrected by the user, it is best to look at the performance in an application-centered manner like error-free letters per minute. But to make the results comparable with the ones presented before, it is worth mentioning that the average accuracy during the free-spelling was 85.4%, which would correspond to an ITR of 115.65 bit/min.

**Table 7:** Results from free-spelling.

Subject	Written	Deleted	Trials	Time [s]	Letters/min	Accuracy
AA	24	6	36	69.57	20.70	83.33%
AC	107	29	165	321.27	19.98	82.42%
AD	88	14	116	224.60	23.51	87.93%
AE	73	11	95	183.37	23.89	88.42%
AG	101	14	129	282.80	21.43	89.15%
AI	34	14	62	118.40	17.23	77.42%
**average**	**71.2**	**14.7**	**100.5**	**200.00**	**21.35**	**85.40%**

Number of written (error-free) letters, number of deleted letters, total number of trials, time in seconds for all trials, average number of error-free letters per minute and average accuracy.

### Reception of the c-VEP BCI by the Subjects

The subjects’ reception of the c-VEP BCI was positive. Although some of them expressed concerns when they first saw the flickering stimuli prior to using it, none of them stated the c-VEP BCI to be annoying when asked at the end of the sessions. None of the subjects reported fatigue or feeling uncomfortable while using the BCI. The three subjects who had previous experience with a P300 Speller, found the c-VEP BCI system more pleasing and stated to prefer using it compared to the P300 Speller.

For the free-spelling condition, the subjects stated that most mistakes were made, because they didn’t find the character in time, but they think that they could increase their accuracy in the free-spelling condition if they would have more time to practice and therefore know the positions of the letters better.

## Discussion

With an average ITR of 136 bit/min during session 1 with unsupervised adaptation and 144 bit/min during session 2 with ErrP-based adaptation this online study shows the potential of a c-VEP BCI to achieve high-performance communication. With previous publications presenting a c-VEP BCI that achieved an average ITR of 108 bit/min [4] and an SSVEP system that showed one subject to reach a peak bitrate of 124 bit/min [19], to the best of our knowledge, the proposed system represents the fastest non-invasive BCI reported to date. It is also notable that 2 subjects achieved an accuracy of 100%, which is remarkable especially when considering the short trial time and the high number of targets.

During the evaluation of the system with free-spelling in a normal-use scenario the subjects were able to write 21.3 error-free letters per minute, which corresponds to an average ITR of 116 bit/min. It has to be noted that the bitrate in the free-spelling is below the results reported for the copy-spelling, which may be attributed to the subjects not finding the correct letter on the matrix in time. Although practice with the system will limit this effect and therefore improve free-spelling performance, the time between trials could also be increased to give the subject additional time to find the letter. Nevertheless results from free-spelling with BCI are scarce in literature and the results presented here show that the proposed system can be used in free-spelling. Despite the performance drop, due to the transfer to free-spelling, the presented system still outperforms all other non-invasive BCI systems.

Regarding the adaptation of the BCI system, the accuracy of the system could be significantly increased from an average of 95% without adaptation to an average accuracy of 96.18% with adaptation based on ErrPs, showing that online adaptation of the BCI improves performance. Although the adaptation based on ErrPs was a little bit better than unsupervised adaptation with 96.05%, it has to be noted that the accuracy with unsupervised adaptation was better for 4 subjects. Since the difference between the results is not statistically significant, it is unclear if adaptation of the BCI profits from the use of ErrPs in the presented system. But we have to point out that through the high general performance of the BCI there is little room for improvement and when comparing the results with unsupervised adaptation and ErrP-based adaptation, it seems that subjects with lower BCI performance tend to benefit more from ErrP-based adaptation, while subjects with higher BCI performance tend to benefit more from unsupervised adaptation. Due to the small subject populations, definitive conclusions regarding this issue cannot be drawn and more studies using ErrP-based adaptation with more low-performing subjects may be needed to further investigate the benefit of ErrP-based adaptation.

Also subject AJ should be mentioned, who was not able to perform a proper calibration session, because she did not see all cues due to excessive blinking caused by her contact lenses. While this shows some restrictions of the system in its current form, we think that these problems could be alleviated by increasing the time of the trials as well as the time of the cues. In addition, multiple sequences could be presented during one trial and the average of these multiple sequences could be used for classification. This method is already successfully used in the P300 Speller [20] and would likewise work in a c-VEP BCI.

### Error-related Potentials

When looking at the ErrPs we found a similar topographic distribution as the ErrPs elicited when using a P300 Speller [8]. But with only two distinct peaks visible in the average ErrP waveform, the shape differs and is missing the first peak at about 270 ms. In addition, the delay of the peaks presented in this paper are about 40 ms less than the delays presented in [8]. This difference may be attributed to the more accurate synchronization of the stimulus by use of the parallel port [21].

Nevertheless the ErrP could be detected with a sufficient average accuracy of 96.7% and a sensitivity of 69.3%, which was sufficient to utilize the ErrP detection for adaptation of the classifier but would also allow to improve performance by an error correction system similar to the one we presented for the P300 Speller [8]. Due to the high accuracy that some subjects achieved, there was very little training data that included ErrPs. Since the amount of trials containing an ErrP negatively correlates with the sensitivity of the classifier we think that the sensitivity could be improved by more training data, i.e., more errors. The negative correlation might also partially explain why the ErrP-based adaptation is having more benefit for subjects with poor BCI performance. As we have shown with the subject-wise cross-validation, the approach of adding ErrP-data from other subjects to increase the amount of training data to improve the sensitivity did not suffice. But this approach could still be used if no ErrP-data is available for a subject.

### ErrP-based Calibration

The results from the ErrP-based calibration show that the presented c-VEP BCI system can be calibrated solely based on the detection of ErrPs, without knowing the true class labels. Only for subject AD the ErrP-based calibration did not work, because ErrPs could not be classified. The reasons for this might be the low number of erroneous trials during session 1. For the subjects where the ErrP detection worked, there was only little deviation from a simulated supervised calibration. Although it is interesting that calibration of the system can be done solely based on the detection of ErrPs, there still needs an application to be found where ErrP-based calibration could be used. One possible benefit could arise, when the c-VEP BCI needs to be restricted to 2 targets, which might be necessary when modifying the stimulus presentation to work without eye-movement control. With 2 targets, an ErrP-based calibration is equal to an ErrP-based adaptation and therewith exists no need to switch between a calibration mode and a use-mode. Assuming the stimulus presentation can be modified to work without eye-movement control, the BCI could be fully operated by paralyzed users without the need for an external person to start calibration-mode or use-mode. In addition, an ErrP-based calibration also allows to transfer information during the calibration, which is not possible with a supervised calibration. However, this benefit might be diminished in the first few trials, due to the low accuracy in the beginning.

### Comparison with Eye-tracker Spellers

Due to the high achieved ITR, also a comparison with spelling applications based on eye-tracking is interesting. In [22] different systems were tested and average writing speeds ranging from 23.5 letters per minute to 54.5 letters per minute were reported for novice users. Although advanced users reached up to 79 letters per minute, the results show that the performance of BCI-based spelling applications (with 21.3 error-free letters per minute) can come in the vicinity of eye-tracker spelling applications. Systems with word completion algorithms can reach even higher typing speeds, but those methods would likewise increase the performance of BCI spelling applications.

### Future Work

While we have shown the proposed system to achieve high-performance communication and it was shown earlier [2] that a c-VEP system with intracranial electrodes can be used by an ALS patient, one main issue with the c-VEP BCI concept is the assumed dependence on eye gaze to control the system. Although it is highly doubtable that a c-VEP BCI with 32 targets can be controlled without eye gaze, there might be a positive outcome when reducing the number of targets. It has been shown that SSVEP BCIs can be controlled without eye gaze [23], [24]. Due to the similarity of a c-VEP BCI with a SSVEP BCI we think that this is also possible for c-VEP BCIs, making the c-VEP BCI usable for paralyzed patients without gaze control. But this needs to be evaluated carefully in a new study.

Regarding the online adaptation of the proposed BCI system a similar approach also needs to be tested with other BCI paradigms and lower-performing subjects to investigate the relationship between the amount of ErrPs in the training data and the sensitivity of the ErrP detection.

### Conclusion

In this paper we have presented a c-VEP BCI that uses online adaptation to improve performance. Adaptation works unsupervised as well as based on ErrPs, although the ErrP-based adaptation has very little benefit compared to the unsupervised adaptation. With an average accuracy of 144 bit/min, the presented c-VEP BCI with ErrP-based adaptation is the fastest non-invasive BCI to date. When the system was tested in free-spelling mode the subjects achieved an average of 21.3 error-free letters per minute, which verifies the feasibility of the presented system in a normal-use scenario and shows that the performance of BCI spelling applications can approach the performance of eye-tracker spelling applications. We have also shown that a calibration of the c-VEP BCI system is possible without having labeled data, solely based on the detection of ErrPs. Despite the current uncertainty if a c-VEP BCI can be used without gaze control we think that the presented system is a valuable step towards faster BCI systems and that the online adaptation is a step towards more robust BCI applications.

## References

[1] Sutter EE (1984) The visual evoked response as a communication channel. In: Proceedings: IEEE Symposium on Biosensors. pp. 85–100.

[2] Sutter EE (1992) The brain response interface: communication through visually-induced electrical brain responses. Journal of Microcomputer Applications 15: 31–45.

[3] Bin G, Gao X, Wang Y, Hong B, Gao S (2009) VEP-based brain-computer interfaces: time, frequency, and code modulations. IEEE Comput Intell Mag 4: 22–26.

[4] Bin G, Gao X, Wang Y, Li Y, Hong B, et al (2011) A high-speed BCI based on code modulation VEP. Journal of Neural Engineering 8: 025015. 21436527 10.1088/1741-2560/8/2/025015

[5] Spüler M, Rosenstiel W, Bogdan M (2012) One class SVM and Canonical Correlation Analysis increase performance in a c-VEP based Brain-Computer Interface (BCI). In: Proceedings of 20th European Symposium on Artificial Neural Networks (ESANN 2012). Bruges, Belgium, pp. 103–108.

[6] Blankertz B, Schafer C, Dornhege G, Curio G (2002) Single trial detection of EEG error potentials: A tool for increasing BCI transmission rates. Lecture notes in computer science: 1137–1143.

[7] Ferrez PW, del R Millán J (2008) Error-related EEG potentials generated during simulated braincomputer interaction. IEEE Trans Biomed Eng 55: 923–929. 18334383 10.1109/TBME.2007.908083

[8] Spüler M, Bensch M, Kleih S, Rosenstiel W, Bogdan M, et al (2012) Online use of error-related potentials in healthy users and people with severe motor impairment increases performance of a P300-BCI. Clinical Neurophysiology 123: 1328–1337. 22244309 10.1016/j.clinph.2011.11.082

[9] Llera A, van Gerven M, Gmez V, Jensen O, Kappen H (2011) On the use of interaction error potentials for adaptive brain computer interfaces. Neural Networks 24: 1120–1127. 21696919 10.1016/j.neunet.2011.05.006

[10] Schalk G, Mcfarland DJ, Hinterberger T, Birbaumer N, Wolpaw JR (2004) BCI2000: A General-Purpose Brain-Computer Interface (BCI) System. IEEE Transactions on Biomedical Engineering 51: 1034–1043. 15188875 10.1109/TBME.2004.827072

[11] Golomb SW (1982) Shift Register Sequences. Laguna Hills, CA: Aegan Park Press.

[12] Bin G, Gao X, Yan Z, Hong B, Gao S (2009) An online multi-channel SSVEP-based braincomputer interface using a canonical correlation analysis method. Journal of Neural Engineering 6: 046002. 19494422 10.1088/1741-2560/6/4/046002

[13] Schölkopf B, Platt C, Taylor SJ, Smola AJ, Williamson RC (2001) Estimating the support of a High-Dimensional Distribution. Neural Computation 13: 1443–1471. 11440593 10.1162/089976601750264965

[14] Chang CC, Lin CJ (2001) LIBSVM: a library for support vector machines. Software available at http://www.csie.ntu.edu.tw/cjlin/libsvm.

[15] Vidaurre C, Sannelli C, Müller KR, Blankertz B (2011) Machine-Learning-Based Coadaptive Calibration for Brain-Computer Interfaces. Neural Computation 23: 791–816. 21162666 10.1162/NECO_a_00089

[16] Wolpaw JR, Birbaumer N, Heetderks WJ, McFarland DJ, Peckham PH, et al (2000) Braincomputer interface technology: A review of the first international meeting. IEEE Transactions on Rehabilitation Engineering 8: 164–173. 10896178 10.1109/tre.2000.847807

[17] Dal Seno B, Matteucci M, Mainardi L (2010) The Utility Metric: A Novel Method to Assess the Overall Performance of Discrete Brain-Computer Interfaces. Neural Systems and Rehabilitation Engineering, IEEE Transactions on 18: 20–28. 10.1109/TNSRE.2009.2032642 20064766

[18] Schlögl A, Keinrath C, Zimmermann D, Scherer R, Leeb R, et al (2007) A fully automated correction method of EOG artifacts in EEG recordings. Clin Neurophysiol 118: 98–104. 17088100 10.1016/j.clinph.2006.09.003

[19] Volosyak I (2011) SSVEP-based Bremen-BCI interface - boosting information transfer rates. Journal of Neural Engineering 8: 036020. 21555847 10.1088/1741-2560/8/3/036020

[20] Farwell LA, Donchin E (1988) Talking off the top of your head: toward a mental prosthesis utilizing event-related brain potentials. Electroencephalogr Clin Neurophysiol 70: 510–523. 2461285 10.1016/0013-4694(88)90149-6

[21] Wilson J, Mellinger J, Schalk G, Williams J (2010) A procedure for measuring latencies in braincomputer interfaces. IEEE Trans Biomed Eng 57: 1785–97. 20403781 10.1109/TBME.2010.2047259 PMC3161621

[22] Urbina MH, Huckauf A (2007) Dwell-time free eye typing approaches. In: Proceedings of the 3rd Conference on Communication by Gaze Interaction (COGAIN 2007): 65–70.

[23] Kelly S, Lalor E, Reilly R, Foxe J (2005) Visual spatial attention tracking using high-density ssvep data for independent brain-computer communication. Neural Systems and Rehabilitation Engineering, IEEE Transactions on 13: 172–178. 10.1109/TNSRE.2005.847369 16003896

[24] Zhang D, Maye A, Gao X, Hong B, Engel AK, et al (2010) An independent brain-computer interface using covert non-spatial visual selective attention. Journal of Neural Engineering 7: 016010. 10.1088/1741-2560/7/1/016010 20083864

